# Supplementary material for: Control of human testis-specific gene expression
Source: PLoS One. 2019 Sep 12;14(9):e0215184. doi: 10.1371/journal.pone.0215184 (PMC6742485; doi:10.1371/journal.pone.0215184)
Supplement: S1 Table — (DOCX) [file pone.0215184.s001.docx]

**S1 Table: Database of human testis-specific genes**

| Index | Gene | Chr | Exp^a^ | CGI^b^ | Promoter | IG^c^ | Testis Cell Type |
| --- | --- | --- | --- | --- | --- | --- | --- |
| 1 | OXCT2 | 1 | 33.5 | Yes | Yes |  |  |
| 2 | SYCP1 | 1 | 21.3 | Yes | Yes |  |  |
| 3 | DMRTB1 | 1 | 58.7 | Yes | Yes |  | spermatogonium |
| 4 | ADAM30 | 1 | 19.8 | Yes | Yes |  |  |
| 5 | C1orf94 | 1 | 19.5 | Yes | Yes |  |  |
| 6 | EPHA10 | 1 | 14.8 | Yes | Yes |  |  |
| 7 | BRDT | 1 | 52 | Yes | Yes |  | spermatocyte |
| 8 | TSACC | 1 | 422.7 | Yes | Yes |  |  |
| 9 | LRRC71 | 1 | 31.5 | Yes | Yes |  |  |
| 10 | AXDND1 | 1 | 9.8 | Yes | Yes |  |  |
| 11 | TDRD5 | 1 | 12.4 | Yes | Yes |  |  |
| 12 | SHCBP1L | 1 | 93.6 | Yes | Yes |  | spermatocyte, spermatid |
| 13 | CCDC185 | 1 | 40.4 | Yes | Yes |  |  |
| 14 | CATSPERE | 1 | 9.2 | Yes | Yes |  | sperm |
| 15 | TTLL10 | 1 | 31.2 | Yes | Yes |  |  |
| 16 | ACTRT2 | 1 | 174.2 | Yes | No | Yes | spermatid, sperm |
| 17 | HORMAD1 | 1 | 45.2 | No | Yes |  |  |
| 18 | RGSL1 | 1 | 7.6 | No | Yes |  |  |
| 19 | SLC9C2 | 1 | 4.4 | No | Yes |  |  |
| 20 | CCDC27 | 1 | 22.3 | No | Yes |  |  |
| 21 | ADCY10 | 1 | 8.3 | No | No |  |  |
| 22 | KLF17 | 1 | 10.9 | No | No |  |  |
| 23 | NUP210L | 1 | 13.9 | No | No |  |  |
| 24 | FAM71A | 1 | 84.9 | No | No |  |  |
| 25 | NBPF4 | 1 | 1.5 | No | No |  |  |
| 26 | APOBEC4 | 1 | 8.1 | No | No |  |  |
| 27 | SMCP | 1 | 743.8 | No | No |  | spermatid |
| 28 | ATP1A4 | 1 | 13.3 | No | No |  |  |
|  |  |  |  |  |  |  |  |
| 29 | TUBA3E | 2 | 162.8 | Yes | No |  |  |
| 30 | MDH1B | 2 | 19.7 | Yes | No |  |  |
| 31 | LINC01191 | 2 | 174.2 | Yes | Yes |  |  |
| 32 | FSIP2 | 2 | 5.7 | Yes | Yes |  |  |
| 33 | BOLL | 2 | 21.5 | Yes | Yes |  | spermatocyte |
| 34 | C2CD6 | 2 | 12.5 | Yes | Yes |  |  |
| 35 | DAW1 | 2 | 21.8 | Yes | Yes |  |  |
| 36 | TSPYL6 | 2 | 18.8 | Yes | Yes | Yes | spermatogonium |
| 37 | SH2D6 | 2 | 5.7 | Yes | Yes |  |  |
| 38 | C2orf78 | 2 | 13.1 | No | No |  |  |
| 39 | LINC01304 | 2 | 14.7 | No | No |  |  |
| 40 | MYADML | 2 | 12 | No | No |  |  |
| 41 | PRR30 | 2 | 170.9 | No | No |  |  |
| 42 | ALS2CR12 | 2 | 11.3 | No | No |  |  |
|  |  |  |  |  |  |  |  |
| 43 | CCDC36 | 3 | 19.2 | Yes | Yes |  |  |
| 44 | TRIM42 | 3 | 19.5 | Yes | No |  |  |
| 45 | DAZL | 3 | 34.1 | Yes | Yes |  | spermatocyte, spermatid, sperm |
| 46 | MORC1 | 3 | 7.7 | Yes | Yes |  | spermatogonium, spermatocyte |
| 47 | ROPN1B | 3 | 60.1 | Yes | Yes |  |  |
| 48 | CCDC37-AS1 | 3 | 60.7 | Yes | Yes |  |  |
| 49 | SPATA16 | 3 | 41.2 | No | No |  |  |
| 50 | TUSC7 | 3 | 6.9 | No | No |  |  |
|  |  |  |  |  |  |  |  |
| 51 | F11-AS1 | 4 | 8.9 | Yes | No |  |  |
| 52 | TKTL2 | 4 | 31.7 | Yes | No | Yes |  |
| 53 | DCAF4L1 | 4 | 21.4 | Yes | No |  |  |
| 54 | CPEB2-AS1 | 4 | 4.4 | Yes | Yes |  |  |
| 55 | ADAD1 | 4 | 67 | Yes | Yes |  |  |
| 56 | SLC25A31 | 4 | 30.5 | Yes | Yes |  | sperm |
| 57 | TTC29 | 4 | 36.7 | Yes | Yes |  |  |
| 58 | RBM46 | 4 | 8.1 | Yes | Yes |  |  |
| 59 | CCDC110 | 4 | 50.3 | Yes | Yes |  |  |
| 60 | GK2 | 4 | 71.3 | No | No | Yes | sperm |
| 61 | C4orf17 | 4 | 8.5 | No | No |  |  |
| 62 | TRIML1 | 4 | 13.3 | No | No |  |  |
| 63 | PDHA2 | 4 | 61.8 | No | No | Yes |  |
| 64 | ADAM29 | 4 | 20.7 | No | No |  |  |
| 65 | CABS1 | 4 | 113.1 | No | No |  | spermatid |
|  |  |  |  |  |  |  |  |
| 66 | C5orf47 | 5 | 18.9 | Yes | Yes |  |  |
| 67 | DDX4 | 5 | 88 | Yes | Yes |  | germ cell |
| 68 | SLCO6A1 | 5 | 18.9 | Yes | Yes |  | Sertoli cell |
| 69 | SLC36A3 | 5 | 6.5 | No | Yes |  |  |
| 70 | C5orf60 | 5 | 31.8 | No | No |  |  |
| 71 | PRDM9 | 5 | 3.8 | No | Yes |  |  |
| 72 | TSSK1B | 5 | 64.1 | No | No | Yes | sperm |
| 73 | MROH2B | 5 | 24.2 | No | No |  |  |
| 74 | FAM71B | 5 | 37.8 | No | No |  | spermatid, sperm |
| 75 | EGFLAM-AS2 | 5 | 5.2 | No | No |  |  |
|  |  |  |  |  |  |  |  |
| 76 | HDGFL1 | 6 | 102.5 | Yes | Yes |  | spermatid |
| 77 | FAM217A | 6 | 17.3 | Yes | Yes |  |  |
| 78 | TCP11 | 6 | 214.6 | Yes | Yes |  | sperm |
| 79 | TCTE1 | 6 | 37 | Yes | Yes |  |  |
| 80 | CFAP206 | 6 | 0.2 | Yes | Yes |  |  |
| 81 | OR2H1 | 6 | 10.9 | No | No |  |  |
| 82 | LINC01015 | 6 | 18.9 | No | No |  |  |
| 83 | PGK2 | 6 | 176.6 | No | No | Yes |  |
| 84 | TTLL2 | 6 | 17.4 | No | No |  |  |
| 85 | C6orf10 | 6 | 35.3 | No | Yes |  |  |
| 86 | CRISP2 | 6 | 554.8 | No | Yes |  |  |
|  |  |  |  |  |  |  |  |
| 87 | PAPOLB | 7 | 16 | Yes | Yes | Yes |  |
| 88 | FKBP6 | 7 | 37.6 | Yes | Yes |  |  |
| 89 | FBXO24 | 7 | 72 | Yes | Yes |  |  |
| 90 | CPA5 | 7 | 7.8 | No | No |  |  |
| 91 | NME8 | 7 | 6.7 | No | No |  |  |
| 92 | SPAM1 | 7 | 8.3 | No | No |  |  |
| 93 | LINC01005 | 7 | 1.3 | No | No |  |  |
| 94 | GALNTL5 | 7 | 16 | No | No |  |  |
| 95 | SEPT14 | 7 | 12.1 | No | No |  |  |
|  |  |  |  |  |  |  |  |
| 96 | DCAF4L2 | 8 | 13.7 | Yes | No |  |  |
| 97 | TEX15 | 8 | 8.1 | Yes | Yes |  |  |
| 98 | ADAM5 | 8 | 9.6 | No | Yes |  |  |
| 99 | KCNU1 | 8 | 5.6 | No | No |  |  |
| 100 | SPATC1 | 8 | 48.6 | No | Yes |  |  |
| 101 | ADAM2 | 8 | 24.3 | No | No | Yes | spermatogenic cell |
| 102 | DNAJC5B | 8 | 29.9 | No | No |  |  |
| 103 | MCMDC2 | 8 | 5.2 | No | Yes |  |  |
| 104 | MROH5 | 8 | 8.3 | No | Yes |  |  |
|  |  |  |  |  |  |  |  |
| 105 | ACTL7B | 9 | 139.8 | Yes | No | Yes | spermatid |
| 106 | DMRT1 | 9 | 21.3 | Yes | Yes |  | spermatogonium |
| 107 | C9orf135-AS1 | 9 | 22.8 | No | No |  |  |
| 108 | C9orf131 | 9 | 39.1 | No | No |  |  |
| 109 | TPD52L3 | 9 | 56.7 | No | No |  |  |
| 110 | FAM205A | 9 | 24.3 | No | No |  |  |
| 111 | ACTL7A | 9 | 255.1 | No | No | Yes | spermatid, sperm |
| 112 | SPATA31C1 | 9 | 6.6 | No | No |  |  |
| 113 | PPP3R2 | 9 | 44.4 | No | Yes | Yes |  |
| 114 | CCIN | 9 | 85.5 | No | No | Yes | sperm |
| 115 | FAM95B1 | 9 | 34.6 | No | No |  |  |
| 116 | SPATA31C2 | 9 | 8.9 | No | No |  |  |
| 117 | SPATA31E1 | 9 | 9.1 | No | No |  |  |
| 118 | ASTN2-AS1 | 9 | 2.5 | No | No |  |  |
|  |  |  |  |  |  |  |  |
| 119 | SPAG6 | 10 | 43 | Yes | Yes |  |  |
| 120 | CCDC7 | 10 | 28.4 | No | Yes |  |  |
| 121 | LINC00838 | 10 | 2.2 | No | No |  |  |
| 122 | LINC00837 | 10 | 1.1 | No | No |  |  |
| 123 | TDRD1 | 10 | 6.5 | No | Yes |  |  |
| 124 | LINC01517 | 10 | 6 | No | No |  |  |
|  |  |  |  |  |  |  |  |
| 125 | CCDC83 | 11 | 19.4 | Yes | Yes |  |  |
| 126 | LDHC | 11 | 166.4 | Yes | Yes |  |  |
| 127 | ACRV1 | 11 | 50.6 | No | No |  | spermatid (acrosome) |
| 128 | UBQLN3 | 11 | 183.1 | No | No |  | sperm |
| 129 | TSGA10IP | 11 | 11.3 | No | No |  |  |
|  |  |  |  |  |  |  |  |
| 130 | H1FNT | 12 | 222.7 | Yes | Yes | Yes | spermatid |
| 131 | TMPRSS12 | 12 | 8.8 | Yes | Yes |  |  |
| 132 | CCDC63 | 12 | 18.5 | Yes | Yes |  |  |
| 133 | CCDC62 | 12 | 28.2 | Yes | Yes |  |  |
| 134 | CCDC38 | 12 | 7.6 | Yes | Yes |  |  |
| 135 | CCER1 | 12 | 42.5 | Yes | No |  |  |
| 136 | GSG1 | 12 | 196.8 | Yes | Yes |  |  |
| 137 | KRT72 | 12 | 24.8 | Yes | Yes |  |  |
| 138 | CEP83-AS1 | 12 | 13.6 | Yes | Yes |  |  |
| 139 | USP44 | 12 | 9.4 | Yes | Yes |  |  |
| 140 | RFX4 | 12 | 43.9 | Yes | Yes |  |  |
| 141 | PIWIL1 | 12 | 39 | Yes | Yes |  | spermatocyte, spermatid |
| 142 | PLCZ1 | 12 | 29.5 | No | No |  | spermatid (acrosome) |
| 143 | OVOS2 | 12 | 14.7 | No | No |  |  |
| 144 | FAM186B | 12 | 42.8 | No | No |  |  |
| 145 | AMHR2 | 12 | 9.4 | No | Yes |  |  |
|  |  |  |  |  |  |  |  |
| 146 | RNF17 | 13 | 15.9 | Yes | Yes |  |  |
| 147 | ANKRD26P3 | 13 | 2.2 | Yes | Yes |  |  |
| 148 | TUBA3C | 13 | 438.6 | Yes | No |  |  |
| 149 | SPERT | 13 | 77.4 | No | No |  |  |
| 150 | OR7E156P | 13 | 5.3 | No | Yes |  |  |
| 151 | LINC00347 | 13 | 6.7 | No | No |  |  |
|  |  |  |  |  |  |  |  |
| 152 | ESR2 | 14 | 2.5 | Yes | Yes |  |  |
| 153 | FSCB | 14 | 35.5 | No | No | Yes | sperm |
| 154 | FAM71D | 14 | 10.9 | No | No |  |  |
| 155 | RPGRIP1 | 14 | 17.6 | No | No |  |  |
| 156 | LRRC74A | 14 | 13.4 | No | Yes |  |  |
| 157 | HEATR4 | 14 | 5.4 | No | No |  |  |
|  |  |  |  |  |  |  |  |
| 158 | LDHAL6B | 15 | 32.8 | Yes | No | Yes | spermatocyte |
| 159 | EXD1 | 15 | 18.1 | Yes | No |  |  |
| 160 | CT62 | 15 | 21.9 | Yes | Yes |  |  |
| 161 | BNC1 | 15 | 15.5 | Yes | Yes |  |  |
| 162 | LINC00930 | 15 | 66.2 | No | No |  |  |
| 163 | GOLGA8S | 15 | 5.1 | No | No |  |  |
| 164 | GOLGA8F | 15 | 1.4 | No | No |  |  |
| 165 | OR4N4 | 15 | 17 | No | No |  |  |
| 166 | NUTM1 | 15 | 22.2 | No | No |  |  |
| 167 | GOLGA8G | 15 | 3.1 | No | No |  |  |
| 168 | TMCO5A | 15 | 31.3 | No | No |  | spermatid (acrosome) |
| 169 | SPATA8 | 15 | 331.7 | No | No |  |  |
|  |  |  |  |  |  |  |  |
| 170 | PRM2 | 16 | 10,134.8 | Yes | No |  | sperm |
| 171 | DPEP3 | 16 | 53.2 | Yes | Yes |  |  |
| 172 | CCDC79 | 16 | 7.8 | Yes | Yes |  |  |
| 173 | FAM57B | 16 | 25.3 | Yes | No |  |  |
| 174 | LINC00917 | 16 | 87.8 | No | No |  |  |
| 175 | LINC00919 | 16 | 49.3 | No | No |  |  |
| 176 | C16orf82 | 16 | 291.2 | No | No | Yes |  |
| 177 | SEPT12 | 16 | 98.8 | No | No |  | sperm |
| 178 | TEKT5 | 16 | 29 | No | No |  |  |
| 179 | CASC16 | 16 | 15.5 | No | No |  |  |
| 180 | OTOA | 16 | 3.9 | No | No |  |  |
| 181 | LINC00254 | 16 | 1.3 | No | No |  |  |
| 182 | PRSS54 | 16 | 31.3 | No | No |  |  |
| 183 | PMFBP1 | 16 | 13.9 | No | No |  |  |
| 184 | PRM1 | 16 | 9670.5 | No | No |  | sperm |
|  |  |  |  |  |  |  |  |
| 185 | MEIOC | 17 | 13.6 | Yes | Yes |  |  |
| 186 | CCDC42 | 17 | 67.9 | Yes | Yes |  |  |
| 187 | TEKT3 | 17 | 28.5 | Yes | Yes |  |  |
| 188 | MYCBPAP | 17 | 51.4 | Yes | Yes |  |  |
| 189 | SPATA22 | 17 | 78.1 | Yes | Yes |  |  |
| 190 | TEX14 | 17 | 21.9 | Yes | Yes |  |  |
| 191 | ZPBP2 | 17 | 70 | Yes | Yes |  | spermatid (acrosome) |
| 192 | MARCH10 | 17 | 49.5 | Yes | Yes |  |  |
| 193 | DNAH9 | 17 | 2.2 | Yes | Yes |  |  |
| 194 | C17orf47 | 17 | 7 | No | No |  |  |
| 195 | CDRT1 | 17 | 1.5 | No | No |  |  |
| 196 | KIF2B | 17 | 79.9 | No | No | Yes |  |
| 197 | HEATR9 | 17 | 5 | No | No |  |  |
| 198 | FBXW10 | 17 | 6.8 | No | No |  |  |
|  |  |  |  |  |  |  |  |
| 199 | CABYR | 18 | 106.4 | Yes | Yes |  |  |
| 200 | CETN1 | 18 | 208.1 | Yes | Yes | Yes |  |
| 201 | CFAP53 | 18 | 87.2 | Yes | Yes |  |  |
| 202 | TXNDC2 | 18 | 65.2 | No | No |  | spermatid |
| 203 | BOD1L2 | 18 | 321.1 | No | Yes |  |  |
|  |  |  |  |  |  |  |  |
| 204 | CCDC114 | 19 | 34.4 | Yes | Yes |  |  |
| 205 | GGN | 19 | 102.3 | Yes | Yes |  |  |
| 206 | CALR3 | 19 | 51.1 | Yes | No |  |  |
| 207 | BIRC8 | 19 | 8.8 | Yes | No |  |  |
| 208 | WDR87 | 19 | 12.9 | Yes | Yes |  |  |
| 209 | NLRP4 | 19 | 4.6 | Yes | No |  |  |
| 210 | HIPK4 | 19 | 17.6 | Yes | No |  |  |
| 211 | DMRTC2 | 19 | 29.9 | Yes | Yes |  |  |
| 212 | RSPH6A | 19 | 30 | Yes | Yes |  |  |
| 213 | ACTL9 | 19 | 120.7 | Yes | No | Yes |  |
| 214 | FAM187B | 19 | 69.2 | No | No |  |  |
| 215 | LINC00906 | 19 | 6.3 | No | No |  |  |
| 216 | NLRP11 | 19 | 1.2 | No | No |  |  |
| 217 | SLC6A16 | 19 | 57.2 | No | No |  |  |
| 218 | SIGLECL1 | 19 | 11.6 | No | No |  |  |
| 219 | LINC00661 | 19 | 37.8 | No | No |  |  |
| 220 | LINC00905 | 19 | 5.1 | No | No |  |  |
|  |  |  |  |  |  |  |  |
| 221 | TTLL9 | 20 | 10.6 | Yes | Yes |  |  |
| 222 | PRND | 20 | 64.8 | No | No |  |  |
| 223 | EPPIN | 20 | 61.4 | No | No |  | sperm |
|  |  |  |  |  |  |  |  |
| 224 | TPTE | 21 | 57.8 | Yes | Yes |  | spermatocyte |
|  |  |  |  |  |  |  |  |
| 225 | HORMAD2 | 22 | 17.5 | Yes | Yes |  |  |
| 226 | RIMBP3 | 22 |  | Yes | Yes | Yes |  |
| 227 | RFPL3S | 22 | 31.5 | No | No |  |  |
| 228 | CCDC116 | 22 | 23.9 | No | No |  |  |
|  |  |  |  |  |  |  |  |
| 229 | RBMXL3 | 23 | 2.9 | Yes | Yes | Yes |  |
| 230 | FAM47B | 23 | 10.5 | Yes | Yes | Yes |  |
| 231 | FAM47C | 23 | 3.3 | Yes | No |  |  |
| 232 | FAM46D | 23 | 1.7 | Yes | Yes |  |  |
| 233 | NXF2B | 23 | 9.5 | No | No |  |  |
| 234 | CYLC1 | 23 | 28.8 | No | No |  | sperm |
| 235 | PASD1 | 23 | 13.7 | No | Yes |  | spermatogonium |
| 236 | MAGEB3 | 23 | 3.7 | No | No |  |  |
| 237 | MAGEB4 | 23 | 7.5 | No | No | Yes |  |
| 238 | MAGEC2 | 23 | 19.8 | No | No |  |  |
| 239 | AKAP4 | 23 | 219.3 | No | No |  | spermatid, sperm |

^a^ RPKM

^b^ CpG island in promoter

^c^ Intronless genes
