# Supplementary material for: Control of human testis-specific gene expression
Source: PLoS One. 2019 Sep 12;14(9):e0215184. doi: 10.1371/journal.pone.0215184 (PMC6742485; doi:10.1371/journal.pone.0215184)
Supplement: S2 Table — (DOCX) [file pone.0215184.s002.docx]

**S2 Table: Human database testis-specific genes with a promoter and a transcription factor binding site near the**

**transcription start site**

| Index | Gene | Chr | TF(s) at Transcription Start Site | Nucleotide Sequence | RE query^a^ |
| --- | --- | --- | --- | --- | --- |
| 2 | SYCP1 | 1 | 1X FOXP3 +5- +10 | GTTGTA | 0.23940 |
| 5 | C1orf94 | 1 | 1X Sp1 -5- +4 | GCCCCGCCCC | 0.01358 |
| 6 | EPHA10 | 1 | 1X c-Ets-1 -6- 0 | CTTCCTC | 0.14159 |
| 7 | BRDT | 1 | 1X TFIID +6- +12 | TTTTGTA | 0.25676 |
| 8 | TSACC | 1 | 1X AP-2αA 0- +5 | GCAGGC | 0.81228 |
| 9 | LRRC71 | 1 | 1X GATA-1 -9- -4; 1X c-Ets-1 +4- +10 | AAGATA, GAGGAAG | 0.14011 |
| 10 | AXDND1 | 1 | 1X RXR-alpha +6- +12 | TTGACCC | 0.28686 |
| 12 | SHCBP1L | 1 | 1X p53 -4- +2 | CCTGCCC | 0.41626 |
| 13 | CCDC185 | 1 | 1X PR A -6- 0 | CACTGTT | 0.06752 |
| 15 | TTLL10 | 1 | 1X Pax-5 -13- -7 | CGAGCCC | 3.16126 |
| 18 | RGSL1 | 1 | 1X IRF-2, 1X TFII-I; -9- -4, -6- -1 | TCACTT, CTTTCC | 0.11886 |
| 31 | LINC01191 | 2 | 1X Pax-5, 1X AP-2αA, -8- -2, -9- -4 | CAGGCCC, CCAGGC | 1.05553 |
| 33 | BOLL | 2 | 1X GR-α, 1X Pax-5, 1X TFII-I -6- +6 | CCTCT, CGCGCCC, CTCTCC | 0.34179 |
| 34 | C2CD6 | 2 | 1X p53 +5- +11 | TCTGCCC | 0.23173 |
| 35 | DAW1 | 2 | 1X Elk-1, -4- +4 | GGCAGGAAG | 0.03542 |
| 36 | TSPYL6 | 2 | 1X Pax-5, +7-+13 | GGGCAGG | 1.39426 |
| 43 | CCDC36 | 3 | 1X RXR-α +7- +13 | GGGTCCG | 0.42973 |
| 45 | DAZL | 3 | 1X c-Ets-1 | TTTCCTG | 0.07315 |
| 55 | ADAD1 | 4 | 1X STAT4, 1X ER-α; +2- +7, +8- +12 | TTTTCC, TGACC | 0.85738 |
| 56 | SLC25A31 | 4 | 1X AP-2αA, -8- -3 | GCAGGC | 0.93730 |
| 57 | TTC29 | 4 | 1X TFII-I, 1X AP-2αA | GGAGAGGCAGGC | 0.27246 |
| 67 | DDX4 | 5 | 1X TFIID | TTTTGAA | 0.22543 |
| 68 | SLCO6A1 | 5 | 1X PR A | GAGTGTT | 0.13667 |
| 69 | SLC36A3 | 5 | 1X Pax-5 +5-+11 | GGGCTCG; | 0.55696 |
| 80 | CFAP206 | 6 | 1X AP-2αA | GGAGGC; | 0.59504 |
| 86 | CRISP2 | 6 | 1X GR-α, 1XTFII-I +4- +14 | ACAGGGAGAG | 0.22445 |
| 87 | PAPOLB | 7 | 1X GR-α, 1X GR-ß 0- +9 | AAAGGAATGG | 2.92442 |
| 88 | FKBP6 | 7 | 1X Pax-5, 1X GCF | CGTGCCC, TGCCCGCGC | 0.88375 |
| 89 | FBXO24 | 7 | 1X AP-2αA | CCAGGC | 1.40892 |
| 97 | TEX15 | 8 | 1XGCF +1- +9 | GCGCGGGGG | 0.64639 |
| 100 | SPATC1 | 8 | 1X NF-AT1, 1X C/EBPα -4- +8 | GGAAAAGAT, GATTGAG | 0.02185 |
| 103 | MCMDC2 | 8 | 1X AP-2αA, +4- +9 | GCAGGC | 0.47234 |
| 104 | MROH5 | 8 | 1X AP-2αA, -9- -4 | GCAGGC | 1.49474 |
| 119 | SPAG6 | 10 | 1X cJun, 1X GR-α -10- 0 | CATGAGTCA, AGAGG | 0.02822 |
| 120 | CCDC7 | 10 | 1X TFII-I -9- -4 | CTTTCC | 0.85461 |
| 123 | TDRD1 | 10 | 1X Pax-5, 1X AP-2αA -1- +6 | GGGCCTGG | 0.75744 |
| 131 | TMPRSS12 | 12 | 1X GR-ß, 1X TBP -1- +12 | CCATT, TTTATAGATT | 0.08266 |
| 132 | CCDC63 | 12 | 1X Sp1 +3- +12 | CGCCCGCCCG | 0.01875 |
| 133 | CCDC62 | 12 | 1X Pax-5 +8- +14 | GGGCGCG | 1.17858 |
| 139 | USP44 | 12 | 2X Pax-5, -8- -2 | GGGCTGG | 2.68945 |
| 145 | AMHR2 | 12 | 1X GR-α 1X TFII-I 0- +6 | GGAAAGG | 0.76089 |
| 147 | ANKRD26P3 | 13 | 1X GRα, 1X STAT4 | ATAGGGGAACT | 3.87875 |
| 150 | OR7E156P | 13 | 1X GR-α, 1X Pax-5 -4- +6 | AGAGGGGCTAG | 1.3338 |
| 152 | ESR2 | 13 | 1X GATA-, 1X C/EBPß; -2- +6 | TATCTGCAA | 0.41198 |
| 160 | CT62 | 15 | 1X Pax-5 -10- -4 | CCCGCCC | 2.84998 |
| 161 | BNC1 | 15 | 1X Pax-5, -8- -2 | GGGCAGG | 3.60264 |
| 185 | MEIOC | 17 | 1X p53, 1X Sp1 +1- +14 | TCTGCCCCGCCC | 0.05035 |
| 189 | SPATA22 | 17 | 1X GR-α, 1X TFII-I -2- +9 | CCTCTTCTCTCC | 0.57862 |
| 190 | TEX14 | 17 | 1X ENKTF1 +5- +12 | CCACGCCA | 0.20719 |
| 191 | ZPBP2 | 17 | 1X NF-1, 0- +9 | TTGGGCCAAG | 0.10149 |
| 204 | CCDC114 | 19 | 1X Pax-5 +9- +15 1XSp1 -4- +16 | CCGGCCCCGAAGCCCCGCCCCT | 0.01483 |
| 211 | DMRTC2 | 19 | 4X GR-ß, -10- -1 | AAATTAATTG | 2.32201 |
| 212 | RSPH6A | 19 | 1X Pax-5 -10- -4, 1X AP-2αA 0- +5 | CGCGCCC, GCCTGC | 1.35518 |
| 224 | TPTE | 21 | 1X PR A, 2X GCF -9- +13 | ACAGTTACCCAGCGCCGGAC | 0.89644 |
| 226 | RIMBP3 | 22 | 1X C/EBP, 1X Pax-5 -4- +3 | CCAAGCCC | 3.27576 |
| 232 | FAM46D | X | 1XHNF-1A 0- +7 | CTTTTAAC | 0.27973 |

^a^ Random expectation; see <http://alggen.lsi.upc.es/cgi-bin/promo_v3/promo/promoinit.cgi?dirDB=TF_8.3>
